# A rare case of unexplained recurrent intracerebral haemorrhage

**DOI:** 10.1093/jscr/rjaf033

**Published:** 2025-02-08

**Authors:** Bo B Cao, Jinlong J Wang, Qinguang Q Ren

**Affiliations:** Department of Orthopedics Center, Affiliated Qingyuan Hospital, The Sixth Clinical Medical School Guangzhou Medical University, Qingyuan People's Hospital, Qingyuan, China; Department of Neurosurgery, Binzhou Medical University Affiliated Shengli Oilfield Central Hospital, Dongying, China; Department of Orthopedics Center, Caoxian People's Hospital, Heze, China

**Keywords:** cerebrovascular disease, recurrent intracerebral haemorrhage, case reports, neurosurgical interventions, rare cerebrovascular cases

## Abstract

Recurrent intracerebral haemorrhage (ICH) presents a complex clinical challenge that eludes traditional diagnostic and treatment approaches. We report a rare and unexplained case of a middle-aged patient with recurrent ICH, for which extensive multidisciplinary investigations have yet to provide a definitive diagnosis or an effective long-term treatment strategy. Intraoperative pathology, genetic testing, cerebral angiography, inflammatory markers (erythrocyte sedimentation rate, C-reactive protein), specific antibodies (ANCA, anti-nuclear antibodies), and immunoglobulin levels showed no specific positive findings. This case highlights the limitations of current diagnostic and therapeutic modalities in recurrent ICH. Further research into the genetic and molecular underpinnings of recurrent ICH is needed to enhance diagnostic accuracy and develop targeted therapies for similar cases.

## Introduction

Recurrent intracerebral haemorrhage (ICH) is a major clinical event, especially in patients with multiple cerebral cavernous malformations [1]. The case described herein involves a female patient with multiple intracranial haemorrhages requiring repeated surgical interventions. Coagulation tests, genetic testing, intraoperative pathology, intraoperative findings, head angiography, head MRI, inflammatory markers, specific and immunoglobulin levels showed no specific positive findings.

Despite the implementation of aggressive treatment measures, the recurrence of bleeding episodes and the gradual decline in neurological function have posed enormous challenges to both the diagnosis and treatment of the patient. Traditional treatment strategies, including surgical and medical therapies, have not been sufficient in halting progression of the disease, and the exact cause of recurrent ICH remains undetermined.

This case study addresses the complexity of managing recurrent ICH in a patient no single identifiable cause. This case analysis aims to present a complex case of cerebral haemorrhage with unexplained recurrent and great distress to the patient and family, we aim to contribute to the broader understanding of recurrent ICH, exploring potential strategies for managing this rare and complex condition.

## Case presentation

The patient is a 58-year-old female with a complex history of recurrent ICH. She has hypertension (grade 1, very high-risk group) and hyperlipidaemia, both of which are currently being treated with medication. Prior to the bleeding episode, the patient had no other significant vascular or neurological diseases in her medical history. There is no family history of cerebrovascular diseases or hereditary vascular malformations. The patient reported a past history of recurrent oral ulcers. The patient’s hypertension has been treated with antihypertensive therapy, including the combined use of enalapril and amlodipine. Her blood pressure is reasonably controlled (112/87 mmHg). The patient's generalized skin and mucous membranes were normal, and the fundus of the eye and sclera showed no obvious abnormalities.

In October 2022, an ICH appeared for the first time in the left parietal lobe, presenting as a dull headache and cognitive impairment primarily manifesting as an inability to recognize familiar Chinese characters but with clear speech (dyslexia), accompanied by slow reactions and impaired memory, calculation, and comprehension abilities and no limb weakness. In March 2023, a second ICH occurred in the left parietal lobe, soon accompanied by a new ICH in the left frontal region, accompanied by cognitive decline, 0/5 muscle strength in the right limbs, increased muscle tone and tendon reflexes in the right limbs, and decreased superficial and deep sensation in the right limbs. In November 2023, subsequent haemorrhages in the left temporal lobe and left cerebellum led to gradual cognitive decline and motor impairment. In May 2024, recurrent ICH emerged in the left parietal lobe and left frontal lobe, accompanied by right limb weakness and cognitive decline. In July 2024, another ICH occurred in the left frontal lobe, whilst another new haemorrhage emerged in the right cerebellum, causing dizziness, nausea, blurred vision, and weakness in the right limbs, along with continued cognitive decline. Despite surgical interventions, the recurrent episodes led to neurological deterioration.

The patient has undergone three craniotomies to evacuate the haematoma and remove the hematoma compressing the brain tissue, stopping the bleeding. Surgical interventions (Video 1) included lesion removal via minimally invasive puncture and haematoma drainage in the left temporal, left parietal, left frontal lobes, and cerebellum, with each procedure targeting the most severe haemorrhage at the time. Postoperatively, neurological recovery was incomplete, and new haemorrhages occurred in different regions of the brain despite the removal of the previous haemorrhagic lesions. After each intervention, the neurological function of the patient deteriorated significantly rather than stabilized.

**Figure 1:**
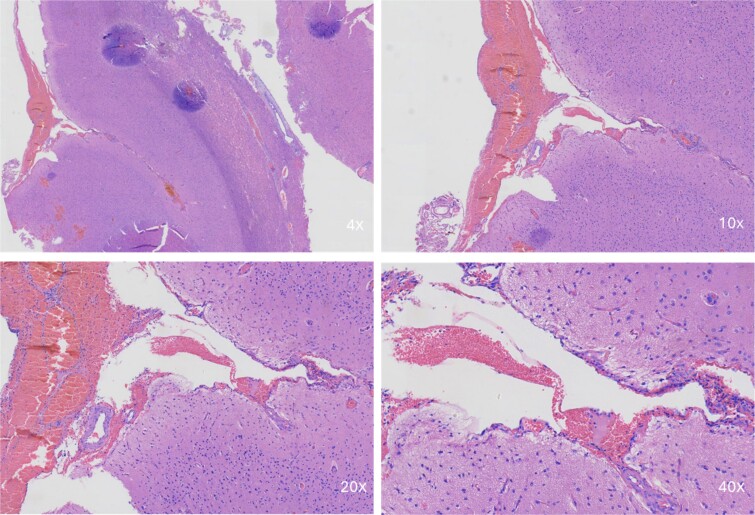
Intraoperative pathological images. Microscopic image showing a portion of the brain tissue with haemorrhagic areas. The vascular morphology in the lesion is irregular, with an indistinct hierarchical vascular wall structure and multiple lumen sizes.

**Figure 2:**
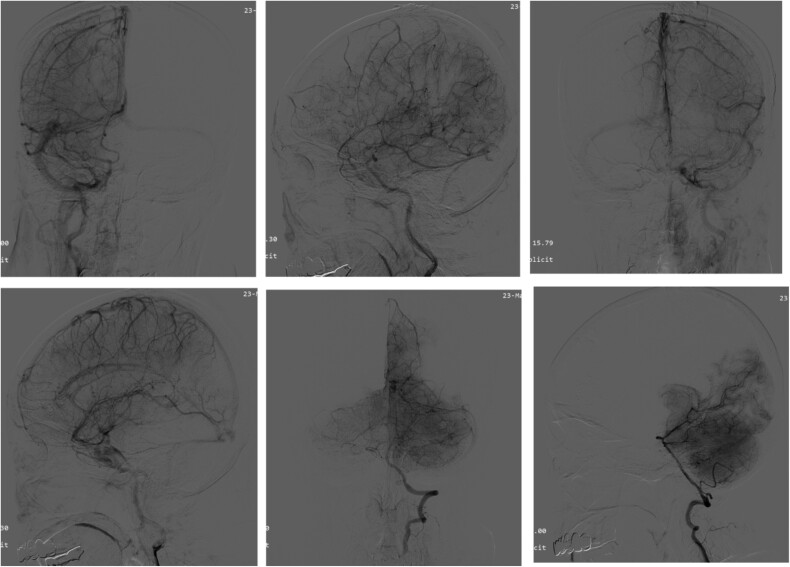
Angiography of the brain. Brain angiography showing obvious vascular malformations.

Intraoperative pathologic images (Fig. 1) showed the vascular morphology in the lesion is irregular, with an indistinct hierarchical vascular wall structure and multiple lumen sizes. Brain angiography (Fig. 2) showed no significant vascular malformations.

**Figure 3:**
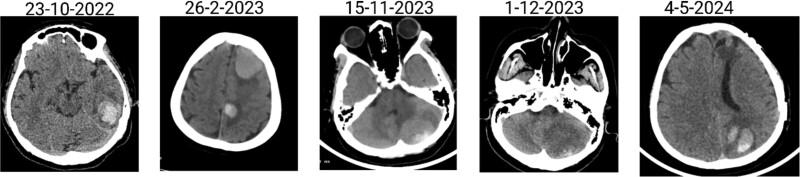
Brain CT scan showing multiple previous haemorrhagic lesions in the brain parenchyma with evidence of chronicity.

**Figure 4:**
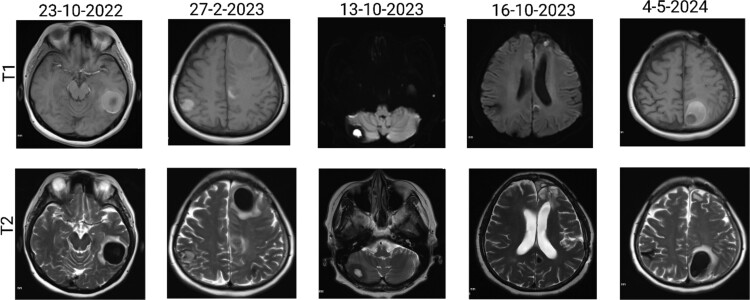
Brain MRI showing dynamic haemorrhage. (a) T2-weighted MR image showing multiple CCMs. The highlighted portions indicate areas of hemosiderin deposition associated with previous haemorrhages. (b) post-contrast T1-weighted MR images showing the enhancement patterns of CCMs and the extent of the surrounding oedema.

**Figure 5:**
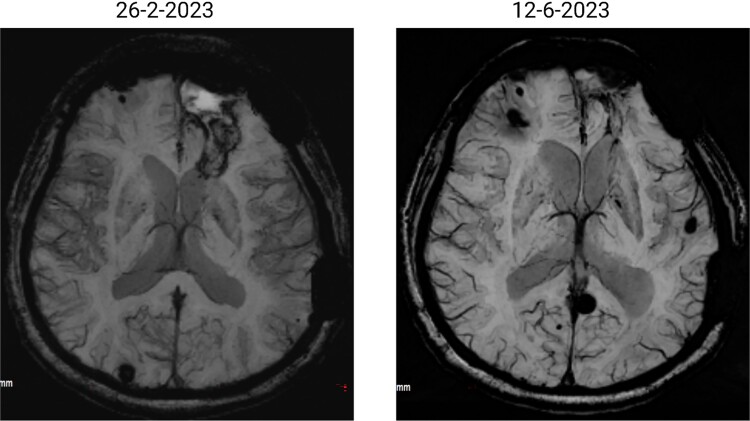
SWI images of the brain. Brain angiography showing obvious vascular malformations. SWI images of the brain showing multiple haemorrhagic lesions with some active bleeding.

The patient has undergone multiple rounds of MRI and CT (Fig. 3) imaging examinations, the results of which have consistently shown a morphology suggestive of recurrent ICH. T2-weighted (Fig. 4) and susceptibility-weighted (SW) MRI (Fig. 5) showed many cerebral cavernous malformations (CCMs) scattered across both cerebral hemispheres, mainly located in the left parietal, frontal, and occipital lobes, with some lesions extending into the brainstem. Multiple lesions presented with a mixed signal intensity corresponding to different stages of haemorrhage and blood degradation products. There was noticeable hemosiderin deposition, indicating the presence of chronic microhaemorrhages. These deposits were particularly extensive in the left parietal lobe, corresponding to the site of the largest and most symptomatic haemorrhage. Regions of gliosis were observed near the haemorrhagic lesions, indicating long-term damage from previous haemorrhages. SWI confirmed the presence of multiple microhaemorrhagic lesions in the parenchyma and subcortical white matter, complicating the clinical picture.

The results (Table 2) from the patient’s coagulation tests (including prothrombin time (PT), activated partial thromboplastin time (aPTT), and international normalized ratio (INR) were all within normal limits. The results from platelet function tests were also normal. The results from extensive autoimmune tests, including antinuclear antibodies (ANAs), antiphospholipid antibodies, and rheumatoid factor, were all negative, excluding vasculitis or autoimmune causes for recurrent haemorrhage. The C-reactive protein (CRP) level and erythrocyte sedimentation rate (ESR) were within normal ranges, and there was no evidence of systemic inflammation. The patient’s lipid profile revealed that the low density lipoprotein (LDL) (Table 1) cholesterol level had increased to 180 mg/dL, with a correspondingly low high-density lipoprotein (HDL) cholesterol level. Triglycerides were also mildly elevated.

**Table 1:** Identified gene mutations.

Gene	Chromosome position	Naming of genetic variants
MME	chr3:155116987	ME:NC_000003. 12(NM_007289.4): c.654 + 1G > A
CLDN11	chr3:170432728	CLDN11:NM_005 602.6:exon3:c.596 C > T:p.P199L
CSF1R	chr5:150078198	CSF1R:NM_00128 8705.3:exon4:c.643 A > G:p.I215V
ITGB3	chr17:47274439	ITGB3:NM_00021 2.3:exon2:c.100C > T:p.R34^*^

**Table 2:** Laboratory test results.

Contents of laboratory tests	Results
Coagulation properties	
PT	12.7 s
aPTT	27.2 s
Platelet count	264 × 10^9^/L
Autoimmune group	
ANA	–
Antiphospholipid antibody	–
CRP	9.35 mg/ml
ESR	20 mm/h
Lipid profile	
LDL	180 mg/dL
HDL	HDL 40 mg/dL
Triglycerides	200 mg/dL
Immunohistochemical results	
GFAP	–
CD34	–
SMA	–

PT: Prothrombin time; aPTT: Activated partial thromboplastin time; ANA: Antinuclear antibody; CRP: C-reactive protein; ESR: erythrocyte sedimentation rate; LDL: low density lipoprotein; HDL: high density lipoprotein.

The patient has undergone extensive genetic testing (Table 1), which has identified mutations in several relevant genes, including MME, CLDN11, CSF1R, and ITGB3. Although these gene mutations are not clearly linked to recurrent CCMs or haemorrhage, their presence raises important questions about the possible genetic predisposition of the patient. Mutations in MME are typically associated with hereditary neurological diseases and may lead to vascular dysfunction, but its specific role in cerebrovascular malformations remains unclear. In this case, the mutations may imply subtle susceptibility to abnormal vascular remodelling or maintenance. CLDN11 (Claudin 11) is involved in the formation of tight junctions in the blood–brain barrier, and its mutation may indicate compromised vascular integrity. Although CCMs have not been clearly associated with CLDN11 gene mutations, it is conceivable that impaired blood–brain barrier functioning may increase this patient’s risk of haemorrhage. The CSF1R gene is associated with adult-onset leukoencephalopathy and can affect microglial function and vascular health. Although CSF1R gene mutations are usually linked to neurodegenerative diseases, the appearance of the mutation in this case may suggest that underlying neurovascular or inflammatory factors are causing the recurrent haemorrhage. ITGB3 gene mutations are associated with platelet function and angiogenesis and may lead to abnormal angiogenesis or impaired vascular stability, potentially contributing to the formation and rupture of cavernous malformations. The combined presence of these gene mutations indicates that cerebrovascular fragility may be a multifactorial genetic susceptibility, although no single gene mutation alone can fully explain the clinical course of the patient. Although familial screening has not been performed, the combination of mutations hints at a possibility of genetic factors.

## Discussion

The case of recurrent ICH in this patient highlights the multifaceted challenges associated with diagnosing and managing complex cerebrovascular conditions, particularly in individuals with multiple contributing factors and no single identifiable cause. Recurrent ICH presents unique difficulties in determining a clear aetiology, accurately assessing haemorrhage risk, and optimizing therapeutic interventions to prevent further events [2]. This discussion elaborates on the potential genetic contributions, explores the limitations of current diagnostic methods, evaluates emerging therapeutic strategies, addresses ethical considerations associated with repeated invasive procedures, compares this case to similar cases in the literature, evaluates the complexities of clinical decision-making, and highlights critical areas for future research to improve the understanding and management of recurrent ICH.

A variety of differential diagnoses for vascular malformations, systemic diseases, cerebral vasculitis, and multiple CCMs have been considered and ruled out [3]. Imaging studies, including CT angiography and MRI, have excluded large vascular malformations such as arteriovenous malformations (AVMs), dural arteriovenous fistulas, and aneurisms [4]. The identified lesions are consistent with CCMs, but their multiplicity and recurrent haemorrhagic nature differ from those of typical isolated cases. Moreover, the patient has undergone comprehensive evaluations to determine whether she has any systemic diseases that could cause vascular fragility, such as coagulation disorders and connective tissue diseases. The results from coagulation and autoimmune tests have been normal, effectively ruling out antiphospholipid syndrome, vasculitis, and other systemic inflammatory or autoimmune diseases. Regarding cerebral vasculitis, in view of the recurrent nature of the haemorrhages, primary cerebral vasculitis has been considered. However, this diagnosis seems unlikely due to the absence of typical angiographic findings and normal inflammatory markers (CRP, ESR).

The current challenge is to determine whether the response for the patient’s recurrent ICH, yet-undetermined systemic processes [5]. Although many common differential diagnoses have been ruled out, the true underlying cause of the patient’s vascular instability remains unresolved [6]. The presence of multiple ICHs poses great diagnostic challenges, especially in determining whether these lesions are a primary manifestation or secondary to an underlying systemic or genetic disease [7]. Sporadic CCMs are typically solitary, whilst the multiple ICHs observed in this case raised suspicions of a genetic or familial predisposition [8].

The identified genetic mutations in MME, CLDN11, CSF1R, and ITGB3 genes raise intriguing possibilities regarding their role in vascular fragility. Although these mutations are not directly associated with ICH in the current literature, emerging evidence suggests that abnormalities in these genes may contribute to endothelial dysfunction, blood–brain barrier instability, or aberrant vascular remodelling. For example, CSF1R mutations are linked to microgliopathy and may alter the neurovascular interface [9], whilst ITGB3 plays a role in platelet aggregation and vascular integrity [10]. Incorporating recent studies on these genes could provide a better understanding of their pathogenic potential and offer insights into targeted therapies. This case underscores the importance of further research into genetic predispositions and their contribution to cerebrovascular diseases.

Comparative analysis of similar cases in the literature demonstrates that recurrent ICH with complex etiologies, such as in patients with multiple ICHs is rare and often lacks standardized treatment guidelines [11]. Murthy *et al.* have documented a range of underlying causes for recurrent ICH, including hypertension, cerebral amyloid angiopathy, and genetic predispositions, particularly in patients with familial syndromes [12]. However, few cases have reported recurrent ICH in the absence of clear genetic or systemic factors, underscoring the unique diagnostic and therapeutic complexities associated with cases of ambiguous aetiology. This case aligns with Karunarathna study in emphasizing the need for a comprehensive and multidisciplinary diagnostic approach [13]. However, it also illustrates a significant limitation in the literature: whilst isolated ICH or genetic syndromes have been studied, there is limited data on multifactorial cases involving patients with both sporadic and familial risk factors, compounded by other potential yet unconfirmed contributors. This gap complicates the generalizability of treatment strategies across diverse presentations of recurrent ICH.

Despite extensive multidisciplinary diagnostics, the exact cause of this patient’s recurrent ICH remains elusive [14]. Conventional imaging modalities, such as MRI and CT angiography, whilst useful, have limitations in detecting microvascular changes or subtle lesions associated with genetic or systemic abnormalities. Many hospitals have provided extensive evaluations, including neuroimaging, genetic testing, and laboratory analyses, yet a unified explanation for recurrent ICH has not been reached [15]. This lack of a unified diagnosis highlights the complexity of this case, wherein traditional diagnostic methods have failed to elucidate the interaction between the various lesions and recurrent bleeding episodes. Emerging technologies, such as ultra-high-resolution MRI and molecular imaging, could offer greater sensitivity in identifying early vascular changes or pinpointing the pathogenesis of recurrent ICH. These tools could also improve the characterization of complex vascular lesions, aiding in both diagnosis and treatment planning. Additionally, the incorporation of artificial intelligence (AI) in imaging analysis holds potential for detecting patterns indicative of rare or multifactorial etiologies.

One of the other most important therapeutic challenges in this case is the determination of the role of repeated surgical resections. The repeated surgical resections performed in this case raise ethical concerns regarding the balance between intervention and the risk of cumulative harm. Surgical morbidity, potential neurological deficits, and diminished quality of life must be weighed against the potential benefits of intervention. Shared decision-making, involving patients and their families, is essential in navigating these challenges. Transparent communication about uncertainties, risks, and alternative management strategies fosters informed consent and aligns care with patient values and preferences. The patient has undergone multiple craniotomies to remove the haemorrhages and resect the ICHs, but the recurrence of the ICH indicates that the surgical interventions did not provide a definitive solution. Every surgery carries inherent risks, including surgical morbidity, potential damage to the surrounding brain tissue, and postoperative complications such as infections, seizures, or further neurological deficits. For multiple ICHs, repeated resection may increase the risk of cumulative brain damage, especially in highly functional regions. Nevertheless, surgical resection remains the primary method for treating asymptomatic CCMs with noticeable haemorrhages or mass effects. In this patient, the decision on whether to proceed with other surgeries is further complicated by the multiplicity of lesions and their recurrence in different locations. Whilst surgery can relieve acute bleeding, it cannot prevent future bleeding caused by other malformations.

Advances in experimental treatments, such as gene-editing technologies like CRISPR and RNA-based therapies, hold promise for addressing underlying genetic susceptibilities in recurrent ICH. Precision medicine, which tailors interventions based on individual genetic and clinical profiles, could revolutionize the management of such complex cases. For instance, vascular-specific genetic modulation or therapies targeting endothelial stabilization could mitigate the recurrence of haemorrhages. Whilst these approaches remain experimental, their potential application to cases like this emphasizes the need for ongoing research and clinical trials to translate these innovations into viable treatments. Regarding hyperlipidaemia, the patient is currently receiving statin therapy to control her elevated LDL levels, but the control effect has been poor. Elevated cholesterol levels may lead to endothelial dysfunction, exacerbating the risk of cerebrovascular events. Although statins are effective in reducing cardiovascular risk, their role in preventing recurrent ICH remains unclear, especially for a patient with underlying vascular malformations. Statins are effective in improving blood vessel function, but they also increase the risk of bleeding [16]. Therefore, traditional therapies for hyperlipidaemia may have a limited efficacy in preventing further haemorrhage; therefore, a more tailored approach to achieving vascular health in this patient is needed.

This case underscores the need for further research to address key knowledge gaps in understanding recurrent ICH, especially in cases with complex or unclear etiologies. Future studies should focus on identifying novel genetic markers and systemic factors that may predispose patients to recurrent haemorrhage, even in the absence of traditional risk factors. Additionally, developing more advanced and specific imaging techniques capable of detecting subtle vascular changes at an earlier stage could provide critical insights into potential causes and facilitate early intervention. Collaborative efforts, including multicentre registries and longitudinal studies, are essential to identify patterns and contributors in similar cases. Advances in genetic research may elucidate novel pathogenic pathways and inform the development of targeted therapies. Additionally, enhancing diagnostic precision through emerging imaging technologies could enable earlier detection and intervention, improving outcomes for patients with complex cerebrovascular conditions. The establishment of multicentre registries for cases of recurrent ICH with atypical etiologies could also improve understanding by providing a larger data set for identifying potential patterns and contributing factors.

Limitations of this study include the single-patient focus, which restricts the generalizability of findings, and the reliance on currently available diagnostic tools, which may not fully capture subtle or emerging risk factors. Without a definitive cause, it remains challenging to offer concrete therapeutic recommendations, and the outcomes observed may not apply to cases with different underlying conditions. Despite these limitations, this case highlights the importance of personalized, multidisciplinary care and calls for a greater emphasis on research to enhance our diagnostic and therapeutic capabilities for managing recurrent ICH.

## Conclusions

This complex case of recurrent ICH underscores the diagnostic and therapeutic challenges inherent in managing patients with repeated haemorrhagic events and unclear underlying etiologies. Key findings include the limited ability of current diagnostic tools to conclusively identify causative factors in multifactorial presentations of ICH, as well as the difficulty in balancing treatment efficacy with risk in the absence of a single, clear aetiology.

## Supplementary Material

222_rjaf033

video_rjaf033

## Data Availability

The authors confirm that the data supporting the findings of this study are available within the article and its supplementary materials. Further enquiries can be directed to the corresponding author.
