# Supplementary material for: A rare case of unexplained recurrent intracerebral haemorrhage
Source: J Surg Case Rep. 2025 Feb 8;2025(2):rjaf033. doi: 10.1093/jscr/rjaf033 (PMC11806917; doi:10.1093/jscr/rjaf033)
Supplement: video_rjaf033 [file video_rjaf033.docx]

**Video. 1** The CCM region is clearly visible under the microscope. This region typically appears red with a soft texture, differing in colour and texture from the surrounding normal brain tissue. Gentle probing with surgical instruments sometimes releases a slow oozing of old blood.
